# Neuritin affects the activity of neuralized-like 1 by promoting degradation and weakening its affinity for substrate: Neuritin regulates the activity of the E3 ligase NEURL1

**DOI:** 10.3724/abbs.2023098

**Published:** 2023-05-29

**Authors:** Jingling Zhu, Yu Li, Chen Zhong, Meiyi Zhu, Yan Zheng, Anying Xiong, Pingping Meng, Liya Shan, Yang Li, Jin Huang

**Affiliations:** 1 Department of Biochemistry and Molecular Biology Tongji Medical College Huazhong University of Science and Technology Wuhan 430030 China; 2 the Key Laboratory of Xinjiang Endemic & Ethnic Diseases and Department of Biochemistry Shihezi University School of Medicine Shihezi 832002 China; 3 the First Affiliated Hospital of Shihezi University School of Medicine Shihezi 832000 China

**Keywords:** neuritin, neuralized-like 1, ubiquitination, degradation, Notch signaling pathway

## Abstract

Neuritin plays a key role in neural development and regeneration by promoting neurite outgrowth and synapse maturation. Our previous research revealed the mechanism by which neuritin inhibits Notch signaling through interaction with neuralized-like 1 (Neurl1) to promote neurite growth. However, how neuritin regulates Notch signaling through Neurl1 has not been elucidated. Here, we first confirm that neuritin is an upstream regulator of Neurl1 and inhibits Notch signaling through Neurl1. Neurl1 is an E3 ubiquitin ligase that can promote ubiquitination and endocytosis of the Notch1 ligand Jagged1. Therefore, we observe the effect of neuritin on the ligase activity of Neurl1. The results indicate that neuritin inhibits Neurl1 activity by reducing the ubiquitination level and endocytosis of the target protein Jagged1. Moreover, we find that decreased activity of Neurl1 results in reduced expression of Notch receptor Notch intracellular domain (NICD) and downstream target gene hairy and enhancer of split-1 ( *HES1*). Furthermore, we investigate how neuritin affects Neurl1 enzyme activity. The results show that neuritin not only weakens the affinity between Neurl1 and Jagged1 but also promotes the degradation of Neurl1 by the 26S proteasome pathway. Taken together, our results suggest that neuritin negatively regulates Notch signaling by inhibiting the activity of Neurl1, promoting the degradation of Neurl1 and weakening the affinity of Neurl1 for Jagged1. Our study clarifies the molecular mechanisms of neuritin in regulating the Notch signaling pathway and provides new clues about how neuritin mediates neural regeneration and plasticity.

## Introduction

Neuritin (CPG15, NRN1) is a neurotrophic factor that plays an important role in nerve development and nerve regeneration [1]. Neuritin proteins are highly expressed during development of the central nervous system and can promote the growth of axons and dendrites, neuron migration and synaptic maturation [ 2, 3]. As such, neuritin can inhibit neuronal apoptosis and maintain the survival of neurons [4]. In addition, after the development and maturation of the central nervous system, the expression of neuritin is closely related to nerve regeneration and repair, learning and memory after injury [ 5– 7]. Furthermore, our previous study showed that recombinant human Neuritin protein [8] can not only promote the regeneration and structural remodelling of peripheral nerve fibres but also accelerate neuromuscular functional recovery after sciatic nerve injury [9]. Moreover, it also plays an important role in the regeneration and repair of central nerve injury-acute spinal cord nerve injury [10]. These findings suggest that neuritin is a promising candidate target for the treatment of nerve injury and neurodegenerative disease. However, the molecular mechanism underlying the effects of neuritin remains obscure.

We previously screened proteins interacting with Neuritin by yeast two-hybrid technology and found that Neuritin could specifically bind to Neurl1. The combination of Neuritin and Neurl1 can inhibit the Notch signaling pathway to promote the outgrowth of neurites [11]. However, how neuritin regulates Neurl1 has not been elucidated. The Neurl1 protein is a RING family E3 ubiquitin ligase located on the cell membrane [ 12, 13] which controls every aspect of eukaryotic biology by promoting protein ubiquitination and degradation [14]. Neurl1 is a positive regulator of Notch signaling in mammals [15]. Research has demonstrated that Neurl1 can promote ligand endocytosis and activate the Notch signaling pathway [ 16, 17] by targeting and ubiquitinating the intracellular domain of substrate Jagged1, which is a single-pass transmembrane protein and a Notch1 ligand [ 18, 19]. These observations prompted us to focus on how neuritin regulates the function of the key enzyme Neurl1. Previous research indicated that E3s are not inflexible structures but rather are active and dynamic enzyme modules whose activities are tightly controlled [20]. Therefore, we hypothesized that neuritin regulates the Notch signaling pathway by affecting the activity of Neurl1. E3 ubiquitin ligase activity is mainly regulated by conformational changes such as phosphorylation, small molecules, substrate competition [ 21, 22] and changes in quantity involving the synthesis and degradation of enzymes [23] .

Here, we report that neuritin inhibits Notch signaling by inhibiting Neurl1 activity. Furthermore, we demonstrate that neuritin not only changes the affinity between Neurl1 and its substrate Jagged1 but also promotes Neurl1 enzyme degradation. This study further clarifies the molecular mechanisms by which neuritin suppresses the Notch signaling pathway.

## Materials and Methods

### Expression plasmids

Recombinant pcDNA3.1-Neuritin (labelled Neuritin), pcDNA3.1-HA-Jagged1 (labelled HA-Jagged1) and pcDNA3.1-Flag-Neuralized (labelled Flag-Neuralized) were constructed as previously described [11]. We amplified the open reading frame sequence of the ubiquitin gene by PCR and inserted it into the pcDNA3.1 expression vector (Invitrogen, Carlsbad, USA) to create a plasmid named PCDNA3.1-ubiquitin, which contains a C-terminal Myc fusion tag. The Myc-labelled full-length ubiquitin expression vector in pcDNA3.1 was provided by Xinjing (Shanghai, China). The constructs of all plasmids were finally identified by DNA sequencing.

### Cell culture and treatment

HEK293 cells were cultured on a regular basis in DMEM supplemented with 4 mM L-glutamine and 4.5 g/L glucose (HyClone, Logan, USA), along with 10% (v/v) fetal bovine serum (Gibco, Carlsbad, USA). The cells were cultured at 37°C under a humidified environment of 95% air and 5% CO _2_ until they reached 70%–80% confluence. Lipofectamine 2000 (Invitrogen) was used to transfect the cells as per the instructions provided by the manufacturer. To analyse the effects of neuritin on the binding affinity of Neurl1 and Jagged1, we explored the optimal transfection dose and time. HEK293 cells were transfected with 0, 200, 300, 400, 500 and 600 ng of pcDNA3.1-HA-Jagged1 to determine the optimal transfection amount for HA-Jagged1, and then cells were transfected with 0, 200, 250, 300, 350 or 400 ng of pcDNA3.1-Flag-Neuralized to determine the optimal transfection amount for Flag-Neuralized. HEK293 cells were then transfected with pcDNA3.1-HA-Jagged1 (600 ng) and pcDNA3.1-Flag-Neuralized (400 ng) for 12, 24, 36 or 48 h to determine the optimal incubation time for *Jagged1* and *Neurl1*. Based on the assigned groups, the cells were transfected with 600 ng HA-Jagged1, 400 ng Flag-Neuralized and 0, 40 or 80 ng of pcDNA3.1-Neuritin for 24 h.

### Immunoprecipitation and western blot analysis

HEK293 cells were transfected by adding the appropriate quantities of plasmid DNA. At 48 h posttransfection, cells were harvested in 0.5 mL of lysis buffer containing protease inhibitors and incubated on ice for 30 min. Immunoprecipitation was carried out as described previously [24], and the lysates were subjected to incubation with agarose beads (GE Healthcare, Chicago, USA) along with their corresponding primary antibodies. Immunoprecipitates were washed three times with washing buffer. The beads were then eluted with elution buffer. Finally, the eluent was detected by western blot analysis.

Western blot analysis was conducted following established protocols [25], and the protein concentration in the supernatants was assessed using the Super-Bradford Protein Assay Kit (CWBIO, Taizhou, China) and subsequently standardized. The protein samples underwent separation via 10% SDS-PAGE and were then transferred onto polyvinylidene difluoride membranes (Millipore, Billerica, USA). Next, the membranes were treated with TBST buffer containing 5% skim milk powder for 2 h to block unwanted interactions. They were then incubated with their respective primary antibodies overnight at 4°C. Then, the membranes were incubated with HRP-conjugated anti-mouse IgG secondary antibody (Sigma-Aldrich, St Louis, USA) at a 1:10,000 dilution after washing with TBST buffer. The bands were visualized by immunoblotting with Chemiluminescent HRP Substrate (Sigma-Aldrich). The sources and dilutions of antibodies were as follows: anti-Flag (1:1000; Sigma-Aldrich) and anti-HA (1:1000; Sigma-Aldrich); anti-Neuritin (1:500; Abcam, Cambridge, UK), anti-Neurl1 (1:2000; Abcam) and anti-NICD (1:2000; Abcam); anti-HES1 (1:1000; Cell Signaling Technologies, Danvers, USA); and anti-β-actin (1:2500; ZSGB-BIO, Beijing, China).

### Indirect immunofluorescence microscopy

HEK293 cells were transfected with different plasmid DNAs in the indicated groups. A total of 2.5×10 ^4^ cells/mL were cultured on slips at 37°C to 50%–60% confluence. The cells were exposed to 4% paraformaldehyde at a temperature of 4°C for 30 min and subsequently incubated in a blocking solution consisting of PBS with 10% (w/v) normal goat serum for 30 min at room temperature. Fixed cells were incubated with primary antibodies diluted in PBS with 1% BSA overnight at 4°C. Detection of the primary antibodies was performed using Alexa Fluor-conjugated secondary antibodies (Sigma-Aldrich). Confocal microscopy was employed to record images with a Zeiss LSM 510 confocal microscope (Carl Zeiss, Oberkochen, Germany). All images were then analysed using ImageJ (NIH, Bethesda, USA).

### Ubiquitination assay

HEK293 cells were transfected with pcDNA3.1-Myc-ubiquitin. After transfection for 48 h, cells were lysed for 15 min in buffer containing 150 mM NaCl, 1% Triton-X 100, 0.5% sodium deoxycholate, 1 M Tris-HCl, pH 7.5, and 1% phenylmethylsulfonyl fluoride (Solarbio, Beijing, China) on ice. The cell lysate was subject to immunoprecipitation (IP) using suitable antibodies that were coupled to Protein G-Sepharose beads (GE Healthcare) for 4 h at 4°C. The resulting precipitates were analysed by western blot analysis with anti-myc antibodies (1:1000; ComWin Biotech, Beijing, China) to determine the level of ubiquitination.

### Protein stability assay

For protein stability assays, HEK293 cells were transfected with Flag-Neuralized and neuritin (untransfected neuritin as control). The cells were seeded at a density of 60%–70% confluence and incubated overnight. After transfection for 40 h, the cells were treated with 80 μg/mL cycloheximide (Sigma-Aldrich) for 0, 2, 4, 6 and 8 h to block the synthesis of proteins. Cells were harvested 48 h after transfection. Protein levels were determined by western blot analysis. To measure how neuritin affects the degradation of Neurl1, cells were treated with proteasome inhibitor (MG132, 10 μM; Sigma-Aldrich) or lysosome inhibitor (NH _4_Cl, 20 μM) for 4 h before harvesting. The expression of Neurl1 was detected by western blot analysis.

### Statistical analysis

All experiments were repeated at least twice with similar results. The western blots were quantified by ImageJ software, and Data are presented as the mean±SEM. *P*<0.05 indicates significant difference.

## Results

### Neuritin regulated Notch signaling via Neurl1

**Figure 1:**
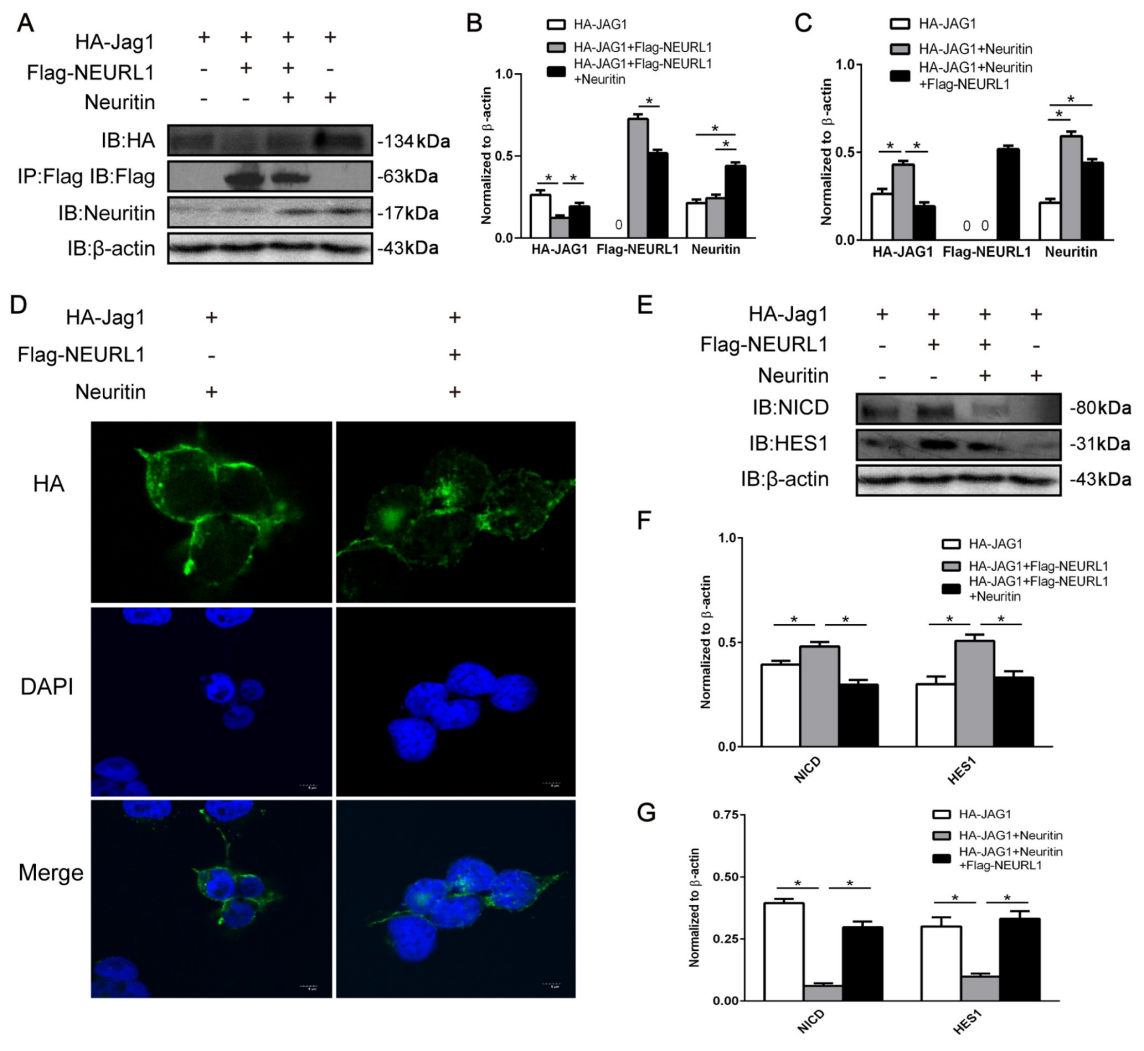
Figure 1 Neuritin regulated Notch signaling via Neurl1 (A–C) Neurl1 reversed the effect of Jagged1 expression by neuritin in HEK293 cells. The expression levels of HA-Jagged1 and other proteins were detected by western blot analysis. Data are presented as the mean±standard error of the mean (SEM) from a minimum of two independent experiments, normalized by β-actin. *P<0.01. (D) Overexpressing Neurl1 rescued the endocytosis of Jagged1. Cells were transfected with neuritin and HA-Jagged1 or with neuritin, HA-Jagged1 and Flag-Neuralized. Jagged1 endocytosis was visualized by immunofluorescence microscopy. Scale bar: 5 μm. (E–G) Overexpression of Neurl1 rescued the inhibition of Notch signaling by neuritin in HEK293 cells. The expression levels of NICD and HES1 were detected by western blot analysis. Data are presented as the mean±SEM from a minimum of two independent experiments; normalized by β-actin. *P <0.01.

Neurl1 facilitates internalization of Jagged1, a ligand of the Notch1 receptor, leading to activation of Notch signaling [16]. Our previous study revealed that neuritin inhibits Notch signaling activation through interaction with Neurl1 [11]. Here, we demonstrated that neuritin regulated Notch signaling via Neurl1. Western blot analysis results showed that the Jagged1 level reduced by Neurl1 was partially increased by overexpression of Neuritin in HEK293 cells, indicating that Neuritin inhibited Jagged1 degradation ( Figure 1A,B). Moreover, overexpression of Neurl1 rescued the Neuritin-induced decrease in degradation of Jagged1 in Neuritin-transfected cells with Neurl1 compared to cells without Neurl1 expression, indicating that Neurl1 is a downstream factor regulated by Neuritin ( Figure 1A,C). These results suggested that neuritin inhibited Neurl1-mediated endocytosis of Jagged1. In addition, immunofluorescence microscopy results showed that endocytosis of Jagged1 was completely restored by overexpression of Neurl1 in HEK293 cells ( Figure 1D). Jagged1 endocytosis is essential for Notch signaling activation [26]. To determine whether neuritin-reduced Jagged1 endocytosis affects Notch activation, we examined the expression levels of NICD and HES1, which are the activated Notch receptor and downstream target gene of Notch signaling, respectively. Western blot analysis results showed that NICD and HES1 were significantly decreased in Neurl1-transfected cells overexpressing Neuritin compared to cells without Neuritin expression ( Figure 1E,F). Similarly, the results showed that Neurl1 was able to rescue the expression levels of NICD and HES1 in Neuritin-overexpressing cells transfected with Neurl1 compared to cells without Neurl1 expression ( Figure 1E,G). These findings suggest that neuritin serves as a regulatory factor for Neurl1 and suppresses Notch signaling via Neurl1.

### Neuritin inhibited the E3 ubiquitin ligase activity of Neurl1

**Figure 2:**
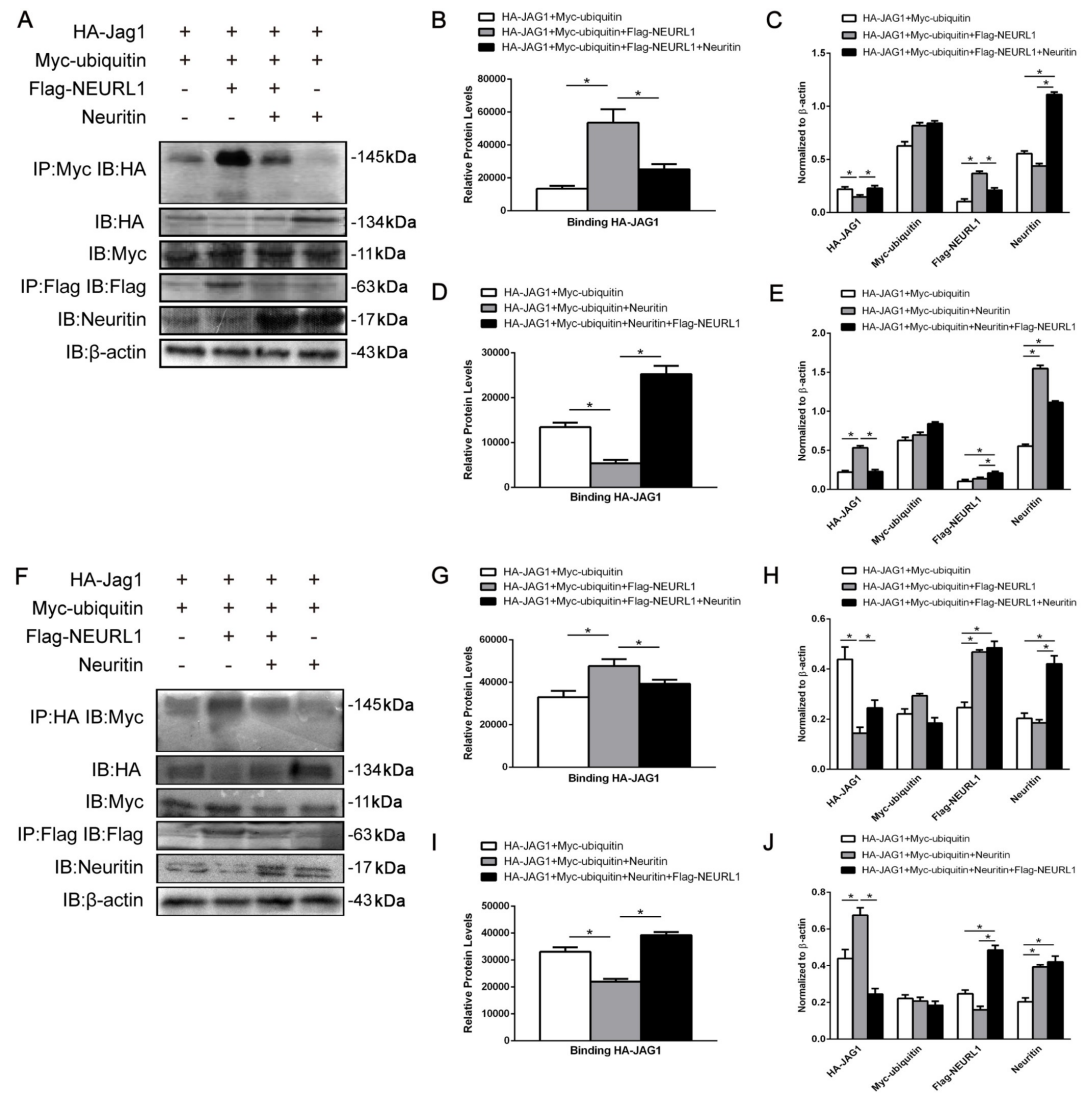
Figure 2 Neuritin inhibited substrate Jagged1 ubiquitination (A–E) Neuritin overexpression reduced the amount of Jagged1 and ubiquitin bound. The quantity of bound Jagged1 and ubiquitin was detected by Co-IP (IP:Myc; IB:HA) in cells expressing HA-Jagged1 and Myc-ubiquitin. (F–J) The quantity of bound Jagged1 and ubiquitin was detected by Co-IP (IP:HA; IB:Myc) in cells expressing HA-Jagged1 and Myc-Ubiquitin. Data are presented as the mean±SEM from a minimum of two independent experiments; normalized by β-actin. *P <0.01.

**Figure 3:**
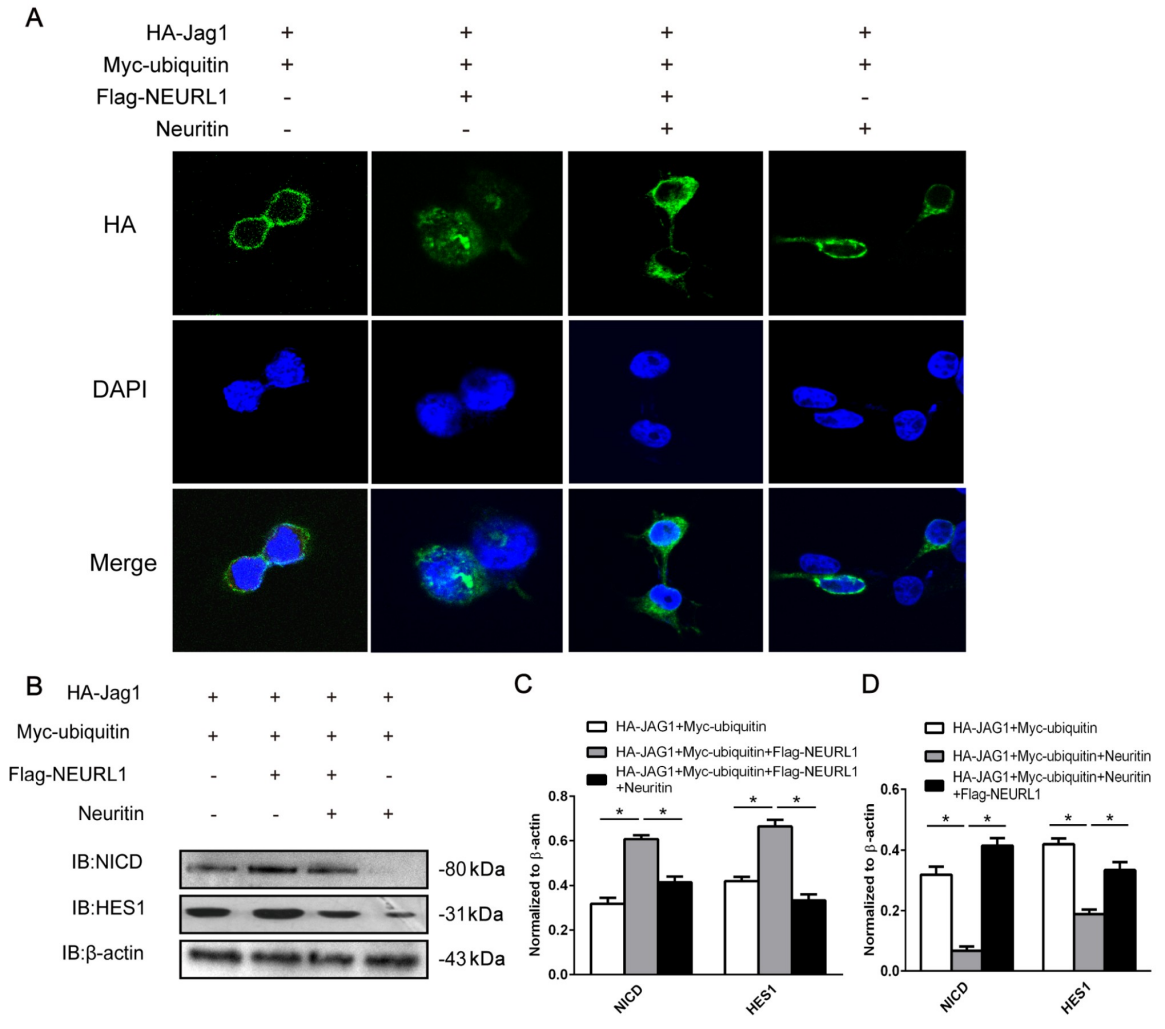
Figure 3 Neuritin inhibited the substrate Jagged1 endocytosis (A) Overexpression of Neuritin suppressed Jagged1 endocytosis mediated by Neurl1 in HEK293 cells expressing HA-Jagged1 and Myc-Ubiquitin. Jagged1 endocytosis was visualized by immunofluorescence microscopy. Scale bar: 5 μm. (B–D) Neuritin suppressed Neurl1-mediated Notch signaling. Cells were transfected with Flag-Neuralized only or with Flag-Neuralized+Neuritin in HEK293 cells expressing HA-Jagged1 and Myc-Ubiquitin. NICD, HES1 and other cellular proteins were detected by western blot analysis. Data are presented as the mean±SEM from a minimum of two independent experiments; normalized by β-actin. *P <0.01.

How does neuritin regulate Neurl1? We first investigated whether neuritin affects Neurl1 function. Neurl1, as an E3 ubiquitin ligase, promotes the ubiquitination of Notch ligands, after which ligand endocytosis activates Notch signaling [16]. We hypothesized that neuritin regulates the Notch signaling pathway by affecting the activity of Neurl1. Therefore, we used coimmunoprecipitation (Co-IP) to detect the ubiquitination level of the substrate Jagged1. The results showed that the quantity of Jagged1 binding to ubiquitin was reduced in Neurl1-transfected cells overexpressing Neuritin compared to cells without Neuritin expression, indicating that overexpression of Neuritin reduced the ubiquitination level of Jagged1 ( Figure 2A,B) and that Neurl1 rescued the effects of Neuritin on reduced ubiquitination in Neuritin-transfected cells expressing Neurl1 compared to cells without Neurl1 expression ( Figure 2A,D). Meanwhile, the results of reverse Co-IP were consistent with the above results ( Figure 2F,G,I). In addition, we observed a single band in the range of Jagged1 ubiquitination (130–170 kDa; nondiffusive state), indicating that Neurl1-mediated substrate ubiquitination is monoubiquitination modification. To test whether neuritin-reduced Jagged1 ubiquitination affects its endocytosis, we used immunofluorescence microscopy to examine the endocytosis of Jagged1. The results showed that overexpression of Neuritin in HA-Jagged1- and Myc-Ubiquitin-transfected cells significantly reduced the Jagged1 endocytosis promoted by Neurl1, whereas Neurl1 could rescue the effects of Neuritin on the reduced endocytosis of Jagged1 in Neuritin-transfected cells expressing Neurl1 compared to cells without Neurl1 expression ( Figure 3A). Finally, to determine whether decreased ubiquitination and endocytosis caused by neuritin affects Notch signaling pathways, we examined the expression levels of NICD and HES1 in HEK293 cells transfected with HA-Jagged1 and Myc-ubiquitin. Western blot analysis results showed that the expressions of NICD and HES1 were reduced in Neurl1-transfected cells expressing Neuritin compared to cells without Neuritin expression ( Figure 3B,C). Moreover, overexpression of Neurl1 rescued the inhibitory effects of neuritin on Notch signaling ( Figure 3B,D). Taken together, the above results indicated that neuritin suppresses the ubiquitination and endocytosis of the substrate Jagged1 by inhibiting the E3 ligase activity of Neurl1, which in turn inhibits the Notch signaling pathway.

### Neuritin weakened the affinity between Neurl1 and substrate Jagged1

**Figure 4:**
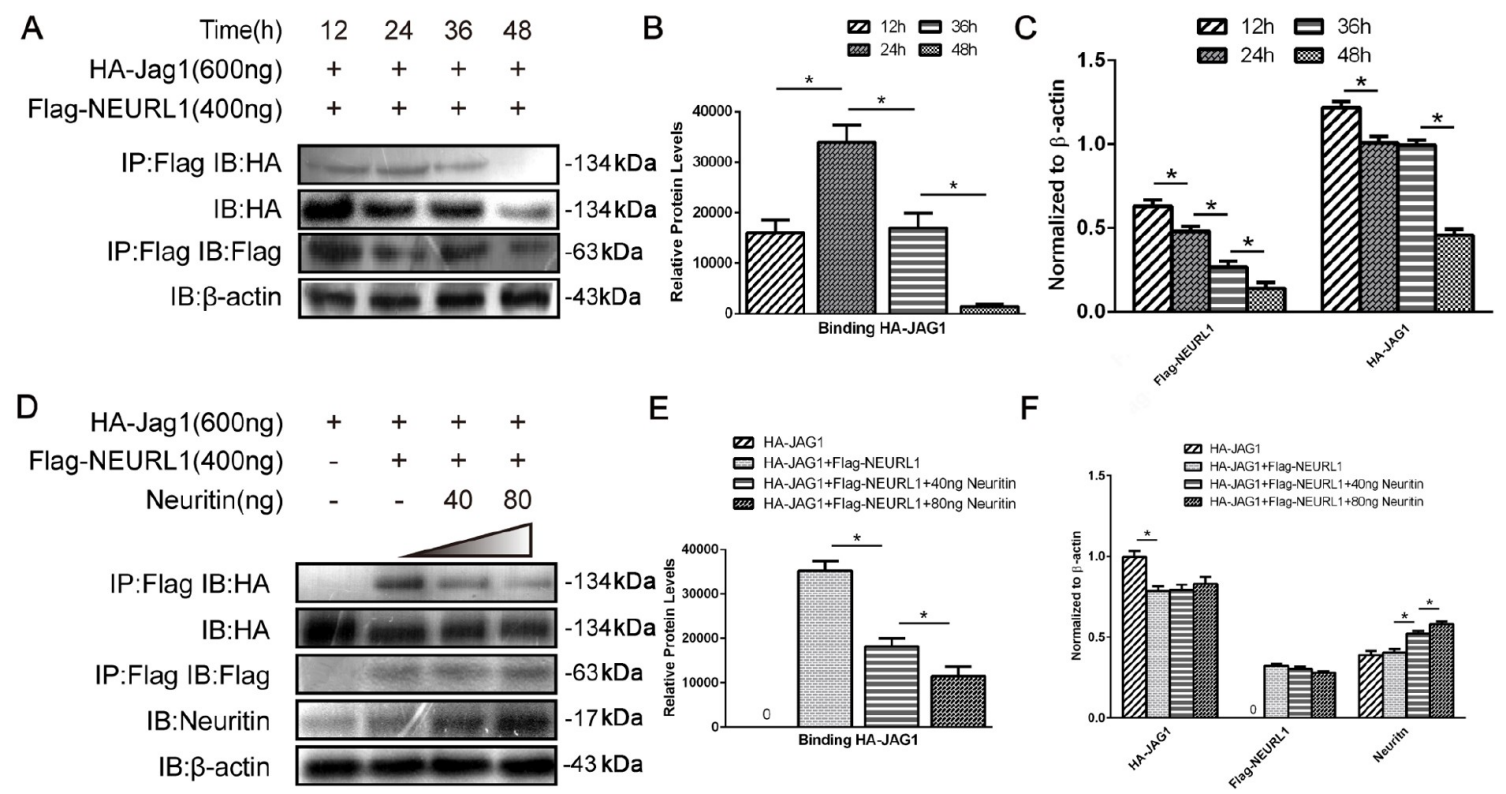
Figure 4 Neuritin weakened the affinity between Neurl1 and Jagged1 (A–C) The optimal binding time points for Jagged1 and Neurl1. HEK293 cells were transfected with HA-Jagged1 and Flag-Neuralized. The proteins were collected after transfection for 12, 24, 36 and 48 h. The binding capacity of Neurl1 and Jagged1 was then evaluated by Co-IP. Data are presented as the mean±SEM from a minimum of two independent experiments; normalized by β-actin. *P<0.01. (D–F) The binding amount of Neurl1 and Jagged1 at different concentrations of neuritin. HEK293 cells were divided into 4 groups. Based on the assigned groups, the cells were transfected with 600 ng HA-Jagged1, 400 ng Flag-Neuralized and 0 ng, 40 ng or 80 ng neuritin. After 24 h, the proteins were collected. Jagged1 protein levels were detected under a concentration gradient of increasing neuritin by IP analysis and western blot analysis. Data are presented as the mean±SEM from a minimum of two independent experiments; normalized by β-actin. *P<0.01.

One of the most important methods for the regulation of key enzymes is conformational change [22]. To explore how neuritin inhibits Neurl1 activity, we used Co-IP to examine the binding quantity of Neurl1 and Jagged1 to evaluate whether neuritin affects their affinity. First, we determined the optimal time for Neurl1 binding to Jagged1. After cells were transfected with Neurl1 for 12 h, 24 h, 36 h and 48 h, total protein was extracted, and the combined quantity of bound Neurl1 and Jagged1 was detected. The results showed that Jagged1 and Neurl1 were bound after 12 h, and the combined bound quantity was increased at 24 h but decreased after 48 h ( Figure 4A,B). Therefore, we determined that the optimum binding time for Neurl1 and Jagged1 was 24 h.

Based on the assigned groups, the cells were transfected with 600 ng HA-Jagged1, 400 ng Flag-Neurl1 and either 0 ng, 40 ng or 80 ng neuritin. After 24 h, co-IP results showed that as the concentration of neuritin increased, the bound quantity of Neurl1 and Jagged1 was gradually decreased, while the expression level of Jagged1 was gradually increased in a dose-dependent manner ( Figure 4D,E). These results indicated that neuritin weakened the binding affinity between Neurl1 and Jagged1, suggesting that neuritin changes the conformation of Neurl1 as an inhibitor molecule.

### Neuritin enhanced the degradation of Neurl1 by the 26S proteasome pathway

**Figure 5:**
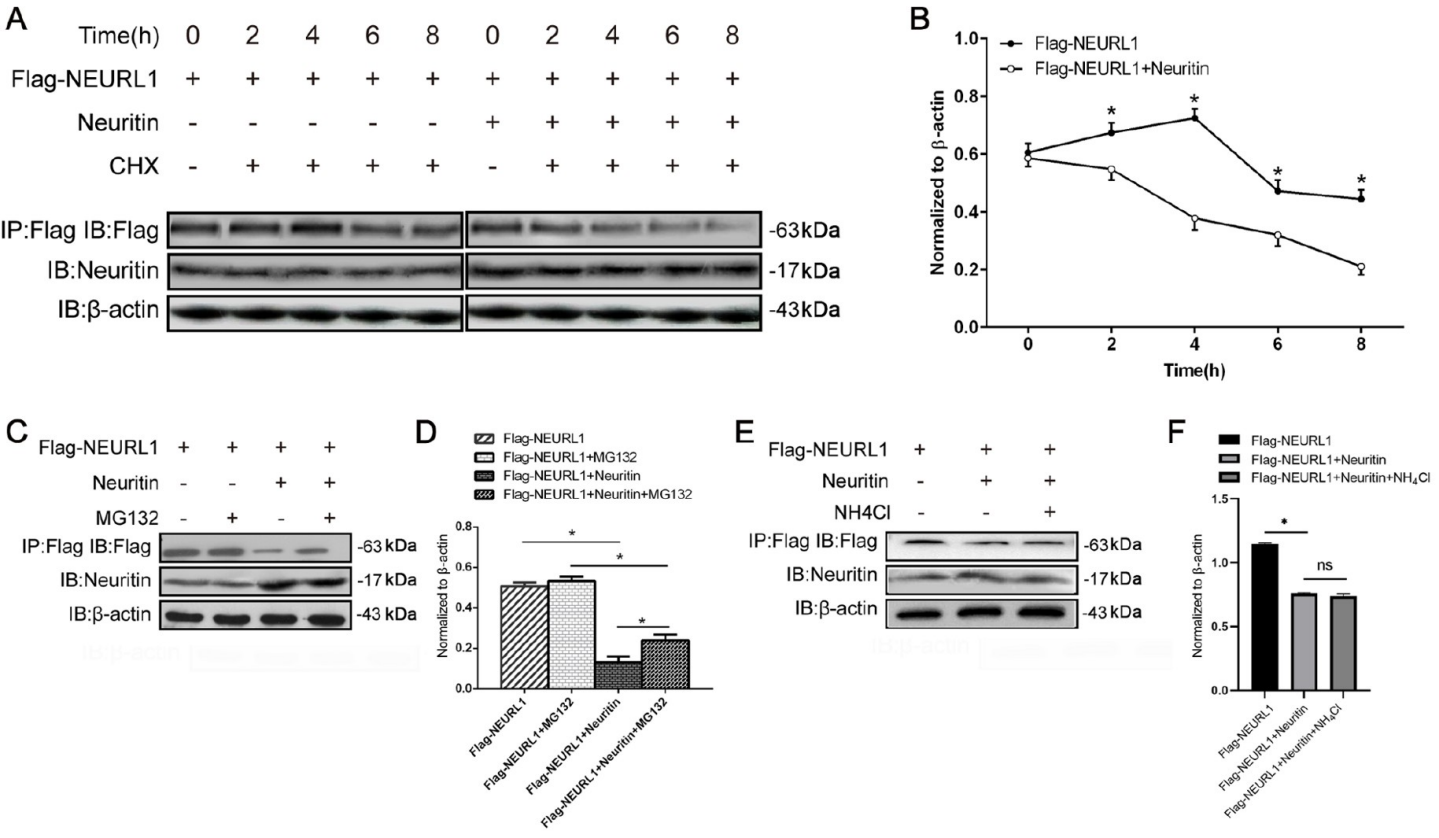
Figure 5 Neuritin enhanced the degradation of Neurl1 via the 26S proteasome pathway (A,B) The effect of neuritin on the stability of Neurl. HEK293 cells expressing Flag-neuralized and transfected with or without neuritin were treated with 80 μg/mL cycloheximide for 0 h, 2 h, 4 h, 6 h and 8 h before being harvested. The expression of Neurl1 was observed over time by western blot analysis. Data are presented as the mean±SEM from a minimum of two independent experiments; normalized by β-actin. *P<0.01. (C–F) The effect of neuritin on the degradation of Neurl. HEK293 cells were transfected with Neurl1 only or with Flag-Neuralized and Neuritin. The cells were treated with MG132 or NH4Cl for 4 h before being harvested, and the expression of Neurl1 was detected by western blot analysis. Data are presented as the mean±SEM from a minimum of two independent experiments; normalized by β-actin. *P<0.01. ns, not significant.

Another way to regulate key enzymes is through the concentration of the enzyme [23]. We used the protein synthesis inhibitor cycloheximide to assess the effect of neuritin on the stability of Neurl1. Western blot analysis results showed that the expression of Neurl1 did not change significantly from 0 to 4 h after cycloheximide treatment and decreased after 6 h in nontransfected control cells. However, the expression of Neurl1 was decreased significantly at 2 h, 4 h, 6 h and 8 h post-cycloheximide treatment in the Neuritin-transfected cells compared to nontransfected cells, indicating that Neuritin markedly accelerated the degradation of Neurl1 ( Figure 5 A,B).

To further investigate how neuritin affects the degradation of Neurl1, we used MG132 to block ubiquitin-dependent 26S proteasome-mediated proteolysis. Western blot analysis results showed that overexpression of Neuritin significantly reduced the expression level of Neurl1 and that MG132 could restore Neuritin-mediated degradation of Neurl1 ( Figure 5C,D). Meanwhile, lysosomal inhibitor (ammonium chloride) did not increase Neurl1 level ( Figure 5E,F). These results indicated that neuritin may degrade Neurl1 through the 26S proteasome pathway.

## Discussion

Neuritin, as a neurotrophic factor, is involved in neural development and nerve regeneration by promoting neurite outgrowth and synapses plasticity. In previous studies, we revealed that neuritin inhibits Notch signaling to promote neurite growth through interaction with Neurl1. Here, we confirmed that neuritin is an upstream and negative regulator of Neurl1 by rescue assays, and neuritin inhibited Notch signaling through Neurl1 ( Figure 1). Nevertheless, the question remains: how does neuritin regulate the E3 ligase Neurl1? In the present study, we mainly studied the effects of neuritin on the activity of the E3 ligase Neurl1.

Neurl1 is an important positive regulator of the Notch signaling pathway [15]. As an E3 ubiquitin ligase, Neurl1 can ubiquitinate the ligand Jagged1 of Notch1 and then promote ligand endocytosis to activate the Notch signaling pathway [ 18, 19]. Therefore, we focused on the regulation of Neurl1 activity by neuritin. We evaluated the activity of Neurl1 affected by neuritin by detecting the ubiquitination level and endocytosis of the target protein Jagged1. The results showed that overexpression of neuritin reduced the ubiquitination level and endocytosis of Jagged1. Additionally, Neurl1 rescued the effects of neuritin on Jagged1 ubiquitination and endocytosis ( Figures 2 and 3A). These results indicated that neuritin inhibited the enzyme activity of Neurl1. Moreover, the decreased activity of Neurl1 resulted in reduced expression of NICD and the downstream target gene *HES1* ( Figure 3B). Therefore, we concluded that neuritin regulates the Notch signaling pathway by inhibiting the activity of Neurl1. In addition, Neurl1-mediated substrate ubiquitination is a monoubiquitination modification [19]. Monoubiquitination is an important regulatory mechanism for proteins *in vivo* that affects endocytosis, gene transcription and nuclear localization [27]. In fact, ubiquitination, and in particular monoubiquitination, has just begun to be appreciated as a signal for endocytosis of transmembrane proteins [28] .

There are a number of different approaches to regulate enzyme activity, including conformation and content regulation [ 22, 23]. To further explore how neuritin regulates Neurl1 activity, we tested whether neuritin acts as an inhibitor to alter the conformation of Neurl1. Our results showed that as the concentration of neuritin increased, the quantity of bound Neurl1 and Jagged1 was gradually decreased over time in a dose-dependent manner ( Figure 4D,E). This finding indicated that neuritin weakened the affinity of Neurl1 and Jagged1 via competitive inhibition. Therefore, neuritin may influence Neurl1 activity by competitively inhibiting the binding of Neurl1 and Jagged1.

In addition, another way to regulate key enzymes is via their quantity. To study the effect of neuritin on the stability of Neurl1, we used cycloheximide to inhibit protein synthesis. Neuritin significantly reduced the stability of Neurl1 and shortened its half-life, indicating that neuritin promoted Neurl1 degradation. Furthermore, we treated the cells with MG132 and ammonium chloride, inhibitors of the proteasome and lysosomes respectively. The results showed that MG132 could restore neuritin-induced increase in Neurl1 degradation, indicating that neuritin enhanced the degradation of Neurl1 via the 26S proteasome pathway ( Figure 5C). It has been reported that unnecessary autoubiquitination and degradation can reduce the functional levels of active E3s [29]. One of the ways to minimize E3 autoubiquitination and maintain E3 stability *in vivo* is through binding to the substrate to prevent its own degradation [ 30, 31] and thus preserve its activity. Therefore, we speculated that neuritin may promote Neurl1 degradation by reducing the affinity between Neurl1 and Jagged1, which ultimately inhibits Neurl1 enzyme activity.

**Figure 6:**
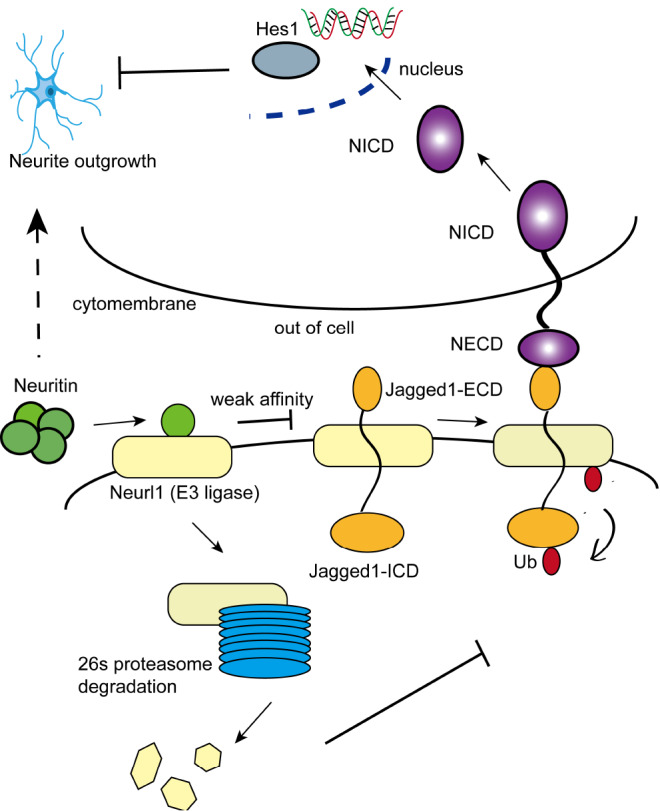
Figure 6 Schematic diagram of neurin inhibition of Neur1 E3 ligase activity The molecular mechanism that neurtin inhibits the E3 ligase activity of Neur1 to inhibit the Notch pathway.

In conclusion, we demonstrated that neuritin inhibits the activity of Neurl1 by weakening the affinity between Neurl1 and Jagged1 and by promoting the degradation of Neurl1 ( Figure 6). Endocytosis of the Notch ligand Jagged1 is then decreased, which inhibits the activation of Notch signaling. This study has thus further clarified the molecular mechanisms underlying neuritin inhibition of the Notch signaling pathway. Our study provides a valuable foundation for further research on neuritin in neural development and neuroplasticity.
